# Supplementary material for: Response of the rare biosphere to environmental stressors in a highly diverse ecosystem (Zodletone spring, OK, USA)
Source: PeerJ. 2015 Aug 20;3:e1182. doi: 10.7717/peerj.1182 (PMC4548494; doi:10.7717/peerj.1182)
Supplement: Table S1 — Unclassified bin denotes the number of sequences that were <85% to any Greengenes database classified entry. Classification was done using the PyNAST pipeline. [file peerj-03-1182-s001.docx]

Supplementary document for:

**Response of the rare biosphere to environmental stressors in a highly diverse ecosystem (Zodletone spring, OK, USA).**

Suzanne Coveley^1^, Mostafa S. Elshahed^1^, and Noha H. Youssef^1*^

^1^Department of Microbiology and Molecular Genetics, Oklahoma State University Stillwater, OK, USA

Table S1. Relative abundance (%) of various bacterial phyla identified in spring sediments, no-stressor control enrichments, as well as salinity and temperature enrichments. Unclassified bin denotes the number of sequences that were < 85% to any Greengenes database classified entry. Classification was done using the PyNAST pipeline (Caporaso et al. 2010).

| Phyla^a^ | Control Unincubated | No-stressor control | Salinity enrichment | | | | | Temperature enrichment | | | |
| --- | --- | --- | --- | --- | --- | --- | --- | --- | --- | --- | --- |
|  |  |  | 1% | 2% | 3% | 4% | 10% | 28ºC | 30ºC | 32ºC | 70ºC |
| Acidobacteria | 1.09 | 1.05 | 0.75 | 1.07 | 0.6 | 0.79 | 0.73 | 1.08 | 1.15 | 0.61 | 0.78 |
| Actinobacteria | 1.897 | 1.54 | 1.79 | 3.57 | 1.85 | 1.92 | 1.71 | 1.4 | 1.501 | 1.5 | 7.94 |
| Armatimonadetes | 0.22 | 0.014 | 0.015 | 0.012 | 0.034 | 0.016 | 0.036 | 0 | 0 | 0.061 | 0.06 |
| Bacteroidetes | 27.98 | 21.04 | 8.76 | 10.52 | 11.34 | 9.1 | 8.71 | 17.42 | 16.33 | 10.86 | 4.14 |
| Caldithrix | 0.29 | 0.007 | 0 | 0.06 | 0 | 0 | 0.018 | 0 | 0 | 0 | 0 |
| Chlorobi | 2.8 | 0.26 | 0.42 | 0.096 | 0.15 | 0.26 | 0.24 | 0.29 | 0.32 | 0.32 | 0.45 |
| Chloroflexi | 4.73 | 1.96 | 1.61 | 4.53 | 1.97 | 1.65 | 2.07 | 1.77 | 1.97 | 2.18 | 2.74 |
| Cyanobacteria | 0.11 | 0.07 | 0.03 | 0.012 | 0.017 | 0.082 | 0.036 | 0.026 | 0.059 | 0.015 | 0.06 |
| Elusimicrobia | 0.076 | 0.007 | 0 | 0 | 0 | 0.016 | 0 | 0.026 | 0 | 0 | 0 |
| Fibrobacteres | 0.66 | 0.07 | 0 | 0.048 | 0 | 0 | 0.054 | 0.053 | 0 | 0 | 0 |
| Firmicutes | 10.75 | 10.04 | 20.34 | 11.55 | 20 | 21.5 | 20.61 | 9.31 | 10.77 | 17.84 | 37.12 |
| Fusobacteria | 0.22 | 0.25 | 0.06 | 0.036 | 0.017 | 0.082 | 0.018 | 0.132 | 0.06 | 0.015 | 0.06 |
| Gemmatimonadetes | 0.495 | 0.17 | 0.18 | 0.9 | 0.19 | 0.25 | 0.47 | 0.37 | 0.41 | 0.21 | 0.28 |
| Gracilibacteria | 2.75 | 0.028 | 0 | 0.048 | 0.034 | 0 | 0.018 | 0.026 | 0 | 0.03 | 0 |
| Lentisphaerae | 0.06 | 0.007 | 0 | 0 | 0 | 0 | 0 | 0 | 0 | 0 | 0 |
| Marinimicrobia | 0.54 | 0.12 | 0.09 | 0.024 | 0.12 | 0.1 | 0.054 | 0.11 | 0.12 | 0.17 | 0.06 |
| Nitrospirae | 0.14 | 0.15 | 0.015 | 0.16 | 0 | 0.033 | 0.13 | 0.21 | 0.09 | 0.015 | 0.11 |
| Aminicenantes | 1.07 | 0.45 | 0.53 | 0.13 | 0.6 | 0.48 | 0.53 | 0.21 | 0.35 | 0.55 | 0.95 |
| Atribacteria | 0.24 | 0.26 | 0.12 | 0.072 | 0.14 | 0.2 | 0.13 | 0.4 | 0.18 | 0.17 | 0.73 |
| Planctomycetes | 0.25 | 0.085 | 0.045 | 0.012 | 0.086 | 0.033 | 0.073 | 0.079 | 0.059 | 0.06 | 0.22 |
| α-Proteobacteria | 3.79 | 2.53 | 6.47 | 6.34 | 6.24 | 5.972 | 5.65 | 2.71 | 2.21 | 6.42 | 5.75 |
| β-Proteobacteria | 1.83 | 1.19 | 1.71 | 1.62 | 1.63 | 1.65 | 1.79 | 1.4 | 1.29 | 1.69 | 0.89 |
| γ-Proteobacteria | 2.78 | 3.66 | 12.29 | 13.17 | 13.25 | 13.91 | 18.24 | 3.24 | 4.67 | 12.13 | 7.86 |
| δ-Proteobacteria | 7.62 | 14.44 | 11.82 | 9.46 | 9.28 | 9.67 | 8.12 | 19.44 | 16.38 | 11.41 | 2.695 |
| ε-Proteobacteria | 1.06 | 1.93 | 1.81 | 1.89 | 2.26 | 1.79 | 1.95 | 1.28 | 1.397 | 2.1 | 0.54 |
| Parcubacteria | 0.403 | 0.036 | 0.03 | 0.12 | 0.034 | 0 | 0.018 | 0.026 | 0 | 0.015 | 0.45 |
| Spirochaetes | 7.496 | 4.04 | 2.31 | 2.42 | 1.94 | 1.59 | 1.89 | 4.1 | 5.44 | 2.12 | 2.91 |
| Synergistetes | 0.61 | 0.11 | 0.045 | 0.012 | 0.051 | 0.08 | 0.054 | 0.11 | 0.29 | 0.076 | 0 |
| Thermi | 0.042 | 0 | 0.015 | 0 | 0 | 0 | 0 | 0.29 | 0.29 | 0.52 | 0.56 |
| Thermotogae | 0.55 | 0.19 | 0.750 | 0.216 | 0.531 | 0.589 | 0.71 | 0.13 | 0.18 | 0.076 | 0.11 |
| TM6 | 0.18 | 0.12 | 0.05 | 0.04 | 0 | 0.13 | 0.073 | 0.13 | 0.18 | 0.091 | 0.06 |
| Saccharibacteria | 0.13 | 0.15 | 0.030 | 0.084 | 0.051 | 0.065 | 0.091 | 0.11 | 0.15 | 0.091 | 0.06 |
| Verrucomicrobia | 0.34 | 0.14 | 0.05 | 0.1 | 0.051 | 0.033 | 0.04 | 0.34 | 0.47 | 0.17 | 0.06 |
| Latescibacteria | 0.22 | 0.31 | 0.2 | 0.23 | 0.14 | 0.18 | 0.13 | 0.026 | 0 | 0.015 | 0 |
| Tenericutes | 3.38 | 0 | 0 | 0 | 0 | 0 | 0 | 0 | 0 | 0 | 0 |
| Other CD | 3.22 | 0.104 | 0.065 | 0.156 | 0.02 | 0.05 | 0.09 | 0.079 | 0.088 | 0.03 | 0 |
| Unclassified | 10 | 33.48 | 27.57 | 34.43 | 27.32 | 27.8 | 25.53 | 33.68 | 33.58 | 28.43 | 22.36 |

a: Gracilibacteria denotes candidate division GN02, Marinimicrobia denotes candidate division SAR406, Aminicenantes denotes candidate division OP8, Atribacteria denotes candidate division OP9, Parcubacteria denotes candidate division OD1, Saccharibacteria denotes candidate division TM7, and Latescibacteria denotes candidate division WS3. Other CD denotes other candidate divisions including SPAM, AD3, CV51, KSB3, NC10, WS6, WPS-2, BRC1, SR1, WS6, ZB3, H-178, GN04, Hyd24-12, OP1, and OP11.

Supplementary figures:

**Supplementary Figure 1.** Rarefaction curves used for calculating diversity rankings shown in Table 1 for temperature (A), and salinity (B) enrichments compared to the no-stressor control. Color coding is as follows: black, no stressor control; purple, temperature incubation at 32ºC or 3% salinity incubation; red, temperature incubation at 28ºC or 1% salinity incubation; green, temperature incubation at 30ºC or 2% salinity incubation; blue, temperature incubation at 70ºC or 4% salinity incubation; and orange, 10% salinity incubation. Rarefaction curves were used for beta diversity ranking where incubations whose rarefaction curve lies on top is considered more diverse and given a higher diversity rank than incubations whose rarefaction curve lie on the bottom.

A

B

**Supplementary Figure 2.** Correlation between enrichment temperature (A) or enrichment salinity (B) and total number of phyla encountered (◇on the primary Y-axis) and rarefaction curve rank (□ on the secondary Y-axis). Pearson correlation coefficients are shown in Table 1.


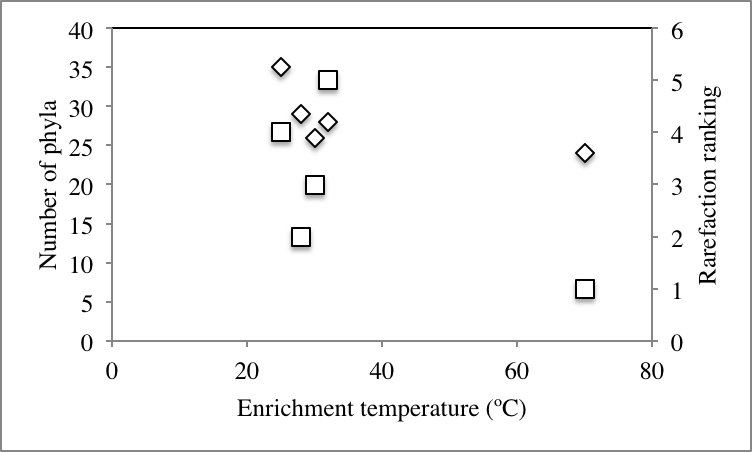

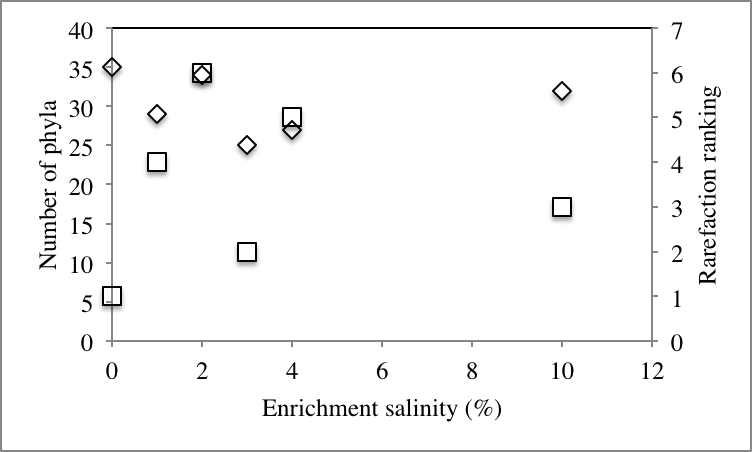


A

B

**Supplementary Figure 3.** Correlation between percentage abundances of various phyla, and the enrichment Temperature (A-B), or salinity (C-D). Phyla whose percentage abundances decreased with the increase in enrichment condition are shown in A-C, while those whose percentage abundances increased with the increase in enrichment conditions are shown in B-D. Symbols: Firmicutes, □; Bacteroidetes, -; Thermotogae, ◇; Latescibacteria (candidate division WS3), ○; Verrucomicrobia, △; Marinimicrobia (candidate division SAR406), 🞩; Aminicenantes (candidate division OP8), 🞵; Parcubacteria (candidate division OD1), 🞤.


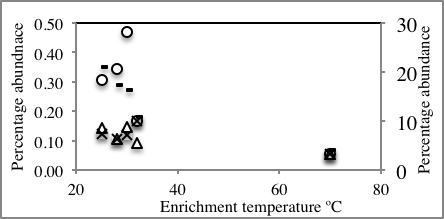

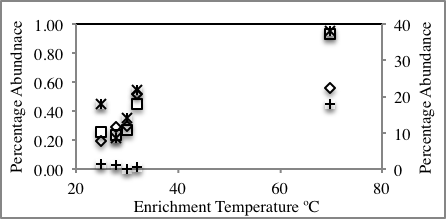

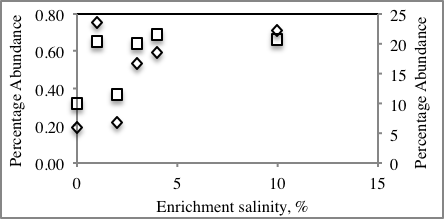

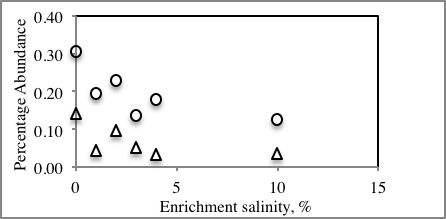


A

B

C

D

**Supplementary Figure 4.** Origins of abundant members in elevated temperature and salinities incubations in various fractions within the no-stressor control incubation. Correlations are shown between the enrichment temperature (A) or salinity (B) on the X-axis, and the percentage of abundant members recruited from abundant members (◇), from unique rare members (□), and from non-unique rare members (△) in the no-stressor control incubation. Abundant and unique rare members contributions to the post-stressor abundant community are shown on the primary Y-axis, while non-unique rare members contribution to the post-stressor abundant community is shown on the primary Y-axis.


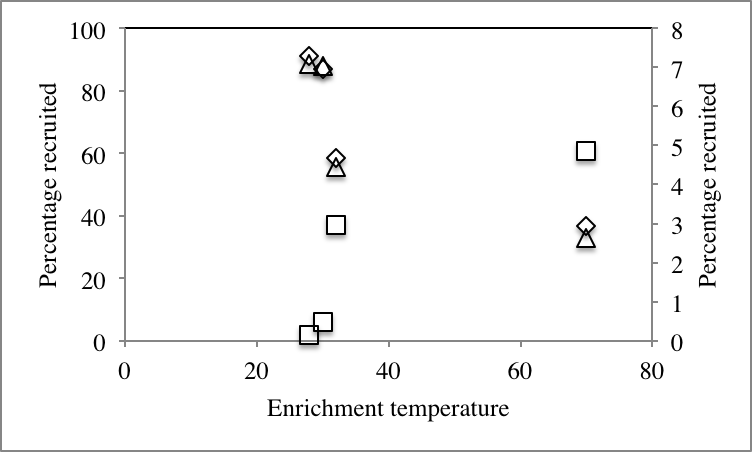

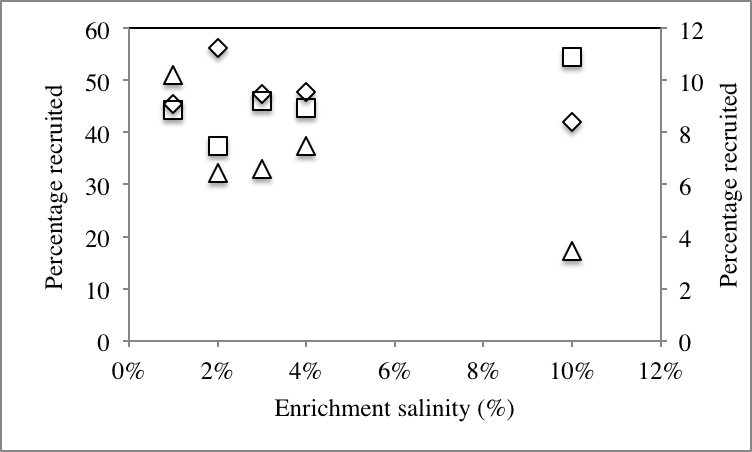


A

B

References.

Caporaso JG, Bittinger K, Bushman FD, DeSantis TZ, Andersen GL, and Knight R. 2010. PyNAST: a flexible tool for aligning sequences to a template alignment. *Bioinformatics* 26:266-267.
