# Supplementary figures and images for: Response of the rare biosphere to environmental stressors in a highly diverse ecosystem (Zodletone spring, OK, USA)
Source: PeerJ. 2015 Aug 20;3:e1182. doi: 10.7717/peerj.1182 (PMC4548494; doi:10.7717/peerj.1182)

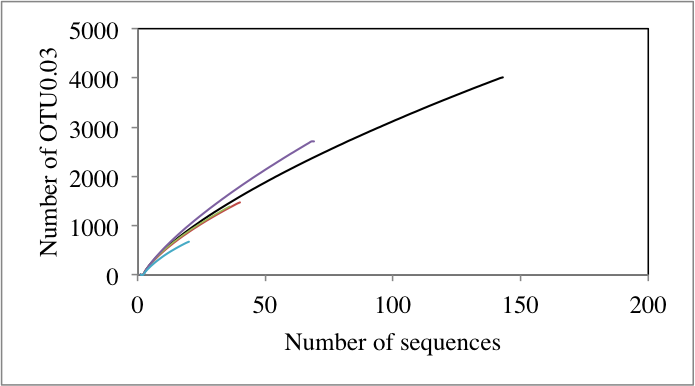

Supplement: Supplemental Information 5 [file peerj-03-1182-s006.png]

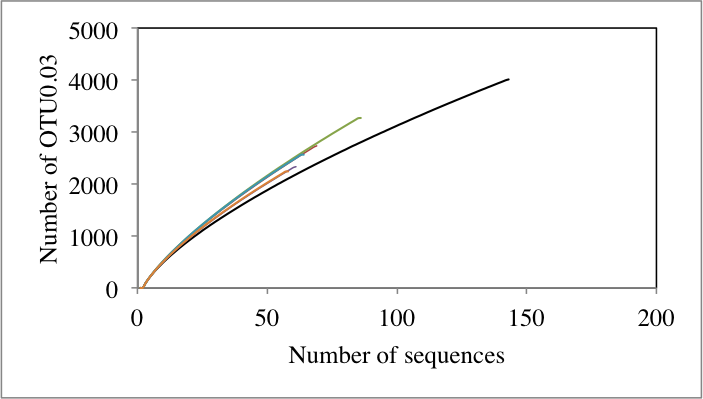

Supplement: Supplemental Information 6 [file peerj-03-1182-s007.png]

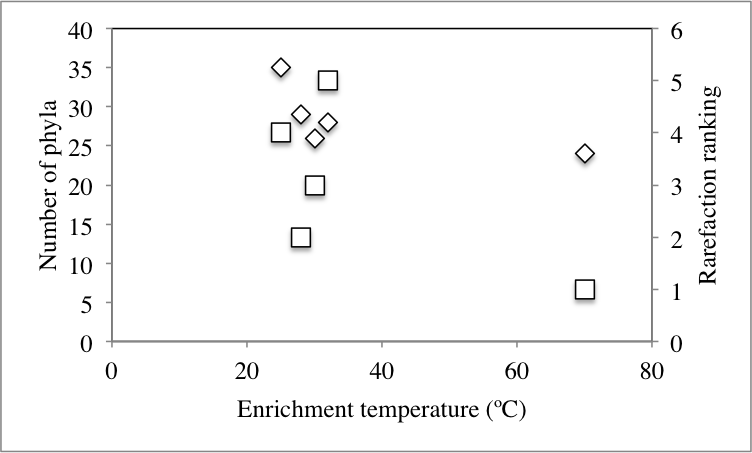

Supplement: Supplemental Information 7 [file peerj-03-1182-s008.png]

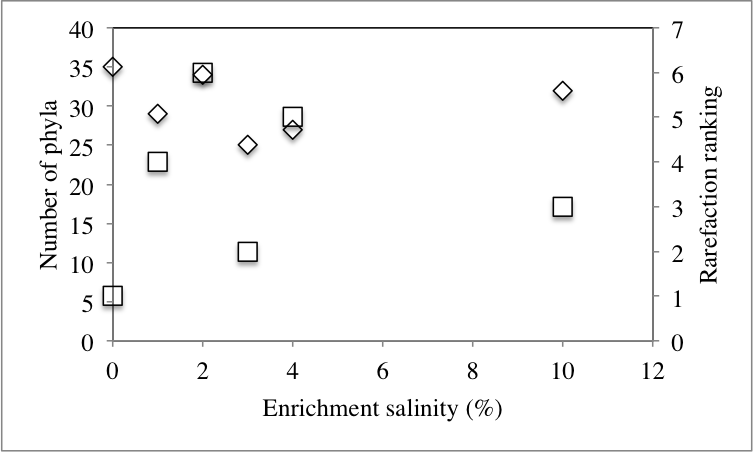

Supplement: Supplemental Information 8 [file peerj-03-1182-s009.png]

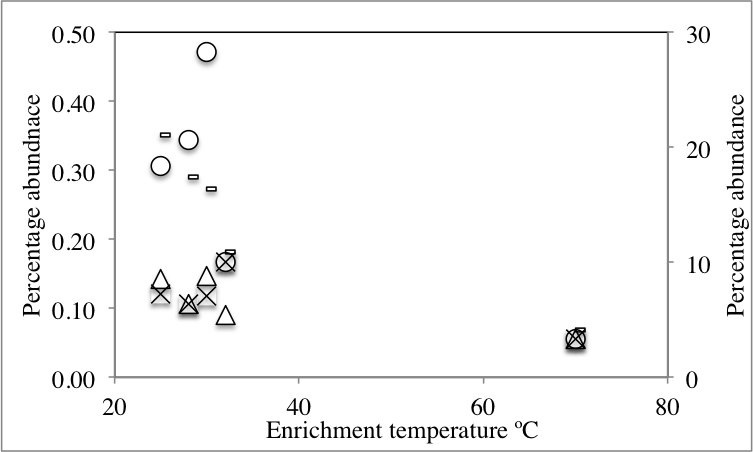

Supplement: Supplemental Information 9 [file peerj-03-1182-s010.png]

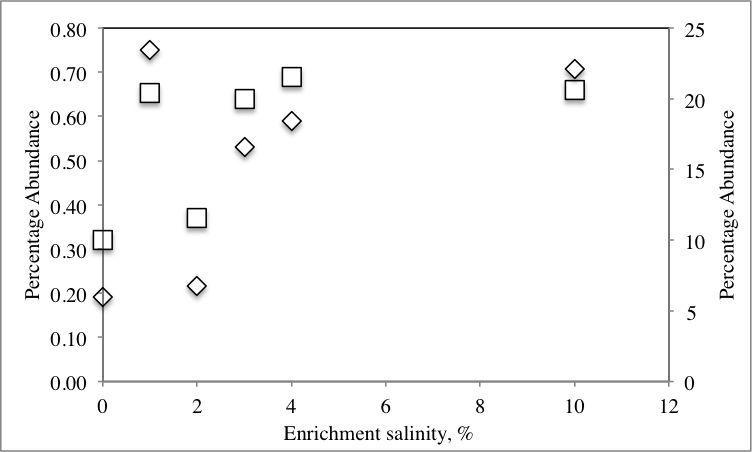

Supplement: Supplemental Information 10 [file peerj-03-1182-s011.png]

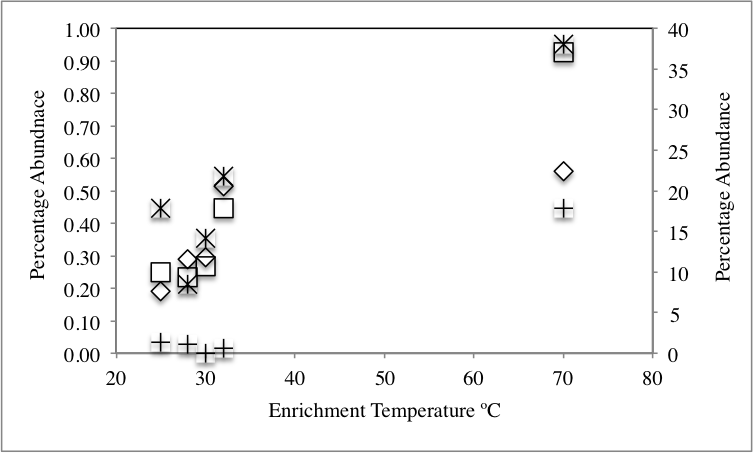

Supplement: Supplemental Information 11 [file peerj-03-1182-s012.png]

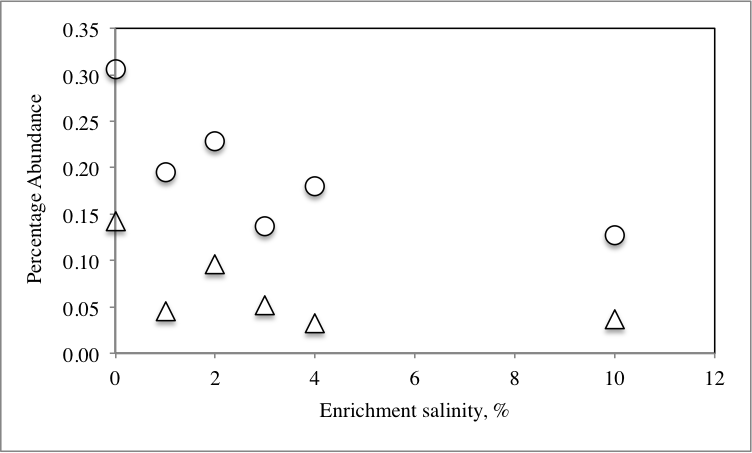

Supplement: Supplemental Information 12 [file peerj-03-1182-s013.png]

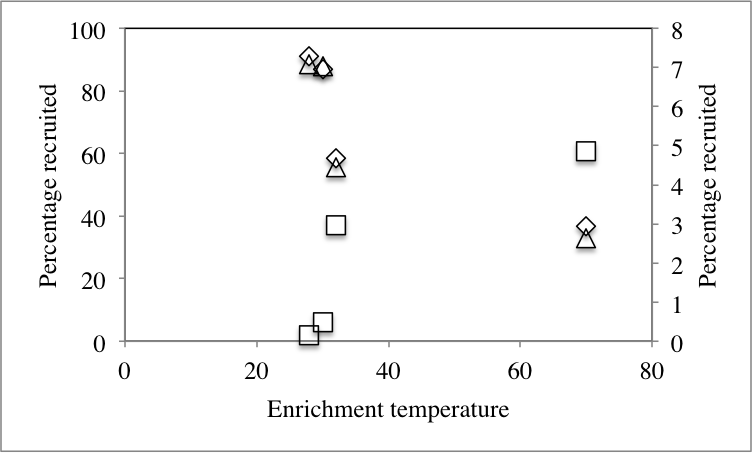

Supplement: Supplemental Information 13 [file peerj-03-1182-s014.png]

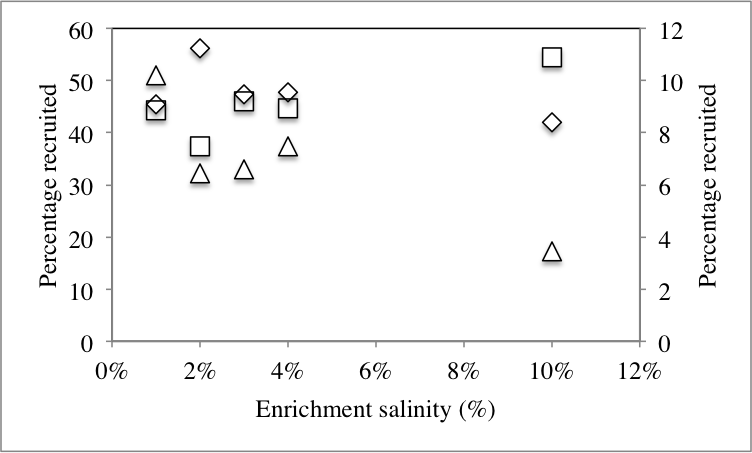

Supplement: Supplemental Information 14 [file peerj-03-1182-s015.png]
